# Biocompatibility Analysis of a Herbal Amalgamation Containing Nochi, Vilvam, and Adhimadhuram Ethanolic Extracts on Vero Cell Line

**DOI:** 10.7759/cureus.77680

**Published:** 2025-01-19

**Authors:** Shalini Govindaraj, Magesh Karuppur Thiagarajan, Swaathi Ravindran, Sathyakumar Mayilvakanam, Aravindhan Ravi, Sivachandran Annamalai

**Affiliations:** 1 Oral Pathology and Microbiology, SRM Kattankulathur Dental College and Hospital, SRM Institute of Science and Technology, Chengalpattu, IND

**Keywords:** aegle marmelos, cell viability, cytotoxicity, ethanolic plant extract, vero cell line, vitex negundo

## Abstract

Objective

This study aimed to examine the biocompatibility and potential synergistic therapeutic effects of a blend of ethanolic extracts derived from the medicinal herbs Vilvam, Nochi, and Adhimadhuram on the Vero cell line.

Materials and methods

The Vero cell line was obtained from the National Centre for Cell Sciences (NCCS), Pune. A herbal formulation was prepared by blending Nochi, Vilvam, and Adhimadhuram ethanolic extracts. The Vero cell line was then evaluated for the effects of the herbal amalgamation at concentrations of 1000 µg, 500 µg, 250 µg, 125 µg, 62.5 µg, 31.2 µg, 15.6 µg, and 7.8 µg with assessments conducted at 24-h and 48-h time points. Untreated cells served as the control.

Results

The herbal elixir exhibited heightened cytotoxicity on the Vero cells, leading to a corresponding decline in the viable cell percentage as the concentration of the formulation was increased. To evaluate the biocompatibility of the herbal consortium on the Vero cell line, a 3-(4, 5-dimethylthiazol-2-yl)-2, 5-diphenyltetrazolium bromide (MTT) assay was carried out over a 24-48-h period of incubation with seven different concentrations as follows: 500 µg, 250 µg, 125 µg, 62.5 µg, 31.2 µg, 15.6 µg, and 7.8 µg. A dose-dependent cell viability was observed. Every experiment was conducted three times.

Conclusion

The herbal formulation showcases impressive biocompatibility. The promising initial results from Vero cell testing represent the first step in a potentially lengthy journey toward clinical application. Vero cell testing demonstrated the herbal blend's exceptional capacity to support cell viability with negligible toxicity, strongly indicating its suitability for future biomedical applications. However, realizing the full potential of this promising herbal consortium will necessitate extensive further research, including rigorous animal studies and clinical trials to meticulously evaluate its safety and efficacy. A deeper investigation into the underlying biocompatibility mechanisms and therapeutic properties will be pivotal to unlocking the transformative possibilities of this innovative herbal blend. Given the burgeoning interest in natural and herbal remedies, this formulation holds tremendous promise; however, its full potential can only be realized through rigorous, systematic scientific evaluation.

## Introduction

In an era where many people seek to minimize their reliance on synthetic pharmaceuticals and explore more natural remedies, the medicinal applications of herbal extracts have garnered heightened interest and attention. Plants have long been a rich source of biologically active compounds, and the untapped wealth of the plant kingdom has increasingly become a target for the search for new drugs and lead molecules for the treatment of various diseases [1]. The use of medicinal plants for health and well-being has deep roots, with evidence suggesting that the practice dates back to ancient civilizations across the globe [2]. Recent scientific investigations have delved deeper into the phytochemical composition and therapeutic potential of a diverse array of herbal extracts, uncovering a wealth of information that has the potential to inform and advance the development of new natural-based treatments. According to data from the World Health Organization (WHO), traditional medicine provides primary healthcare to over 80% of the global population [3,4]. Nonetheless, a lot of people in developing nations, particularly in Asia and Africa, continue to use unrefined herbal extracts to treat a variety of illnesses in both humans and animals [5,6].

*Vitex negundo*, locally known as "Nochi" in Tamil language, commonly referred to as the "chaste tree," a shrub native to Asia, has long been celebrated for its remarkable medicinal properties. The leaf extract of this plant has garnered significant attention in the scientific community due to its diverse pharmacological activities [7]. *Aegle marmelos *(*A. marmelos*), a tree indigenous to the Indian subcontinent, is widely recognized for its diverse array of medicinal applications [8]. The tree, commonly known as the "Bael" or "Wood Apple," belongs to the family Rutaceae and is found across various parts of Asia, including India, Bangladesh, Burma, Pakistan, Sri Lanka, and Thailand [9]. Every part of this plant, from its fruits and leaves to its stem and root bark, has been valued for its unique phytochemical constituents and therapeutic potential [9]. Beyond its potential as an anticancer agent, *A. marmelos* leaf extract has also been investigated for its ability to treat other health conditions. This remarkable compound has demonstrated formidable antimicrobial as well as antiviral capabilities, rendering it a highly promising therapy for combating infectious diseases [10]. Little perennial herb *Glycyrrhiza glabra*, known as "Adhimadhuram" in Tamil, commonly referred to as licorice, has long been used to cure a wide range of illnesses. One of the key bioactive compounds found in *Glycyrrhiza glabra* leaves is glycyrrhizin, a triterpene glycoside. Glycyrrhizin is known to exhibit anti-inflammatory, antiulcer, and antihepatotoxic properties [11]. Furthermore, the existence of additional phenolic substances, including flavonoids and phenolic acids, has been linked to the leaves' strong antioxidant properties [12]. Employing a combination of herbal extracts may offer distinct advantages over utilizing a single extract. These complex plant-derived mixtures harbor a wealth of bioactive compounds that can interact in sophisticated ways, potentially unlocking synergistic benefits that could enhance the overall therapeutic efficacy while minimizing the risks of adverse interactions [13]. Leveraging a blend of herbal extracts empowers researchers and clinicians to unlock synergistic benefits, where the collective impact of varied phytochemicals can amplify therapeutic efficacy while also minimizing the risk of adverse interactions [14].

Ethanolic extraction is an advantageous technique for preserving the potency and integrity of the extracted plant compounds. Ethanol, a mild- and non-polar solvent, can efficiently solubilize a range of lipophilic and hydrophilic phytochemicals without causing substantial degradation or structural alterations. Hence, this study intends to examine the biocompatibility and potential synergistic therapeutic effects of a blend of ethanolic extracts derived from the medicinal herbs Vilvam, Nochi, and Adhimadhuram on the Vero cell line. Investigating this herbal combination could provide invaluable insights to progress the development of safe and effective plant-based healthcare solutions [15].

## Materials and methods

Extract preparation

Fresh leaves (Nochi, Vilvam, Adhimadhuram) were directly sourced from the trees in Tamil Nadu. After a week of air drying in the shade, the gathered plant material was crushed with a mortar and pestle to a powder form. The airtight containers were used to store the pulverized samples for subsequent analysis. Twenty-five grams of each sample were individually extracted with 300 mL of 70% ethanol using a Soxhlet apparatus for 10 h. A rotary evaporator running at 50°C with lowered pressure was then used to collect and concentrate the extractant after it had been removed from the apparatus. Cool storage was provided for the resulting crude extract, which was made with 70% ethanol at a concentration of 100 mg/mL. Equal proportions of aliquots of 1 mg/mL were collected from each sample and combined to prepare the consortium. The consortium was further analyzed to obtain a comprehensive understanding of the plant-based compounds present in the samples.

Biocompatibility

Cell Line and Culture

The Vero cell line was acquired from the National Centre for Cell Sciences (NCCS), Pune. Penicillin (100 μg/mL), streptomycin (100 μg/mL), and 10% fetal bovine serum (FBS) were added to Dulbecco's Modified Eagle's Medium (DMEM) at 37°C in a humidified environment with 50 μg/mL carbon dioxide (CO_2_).

In Vitro Biocompatibility 3-(4,5-dimethyl-2-yl)-2,5-diphenyltetrazolium bromide (MTT) Assay

**Figure 1:**
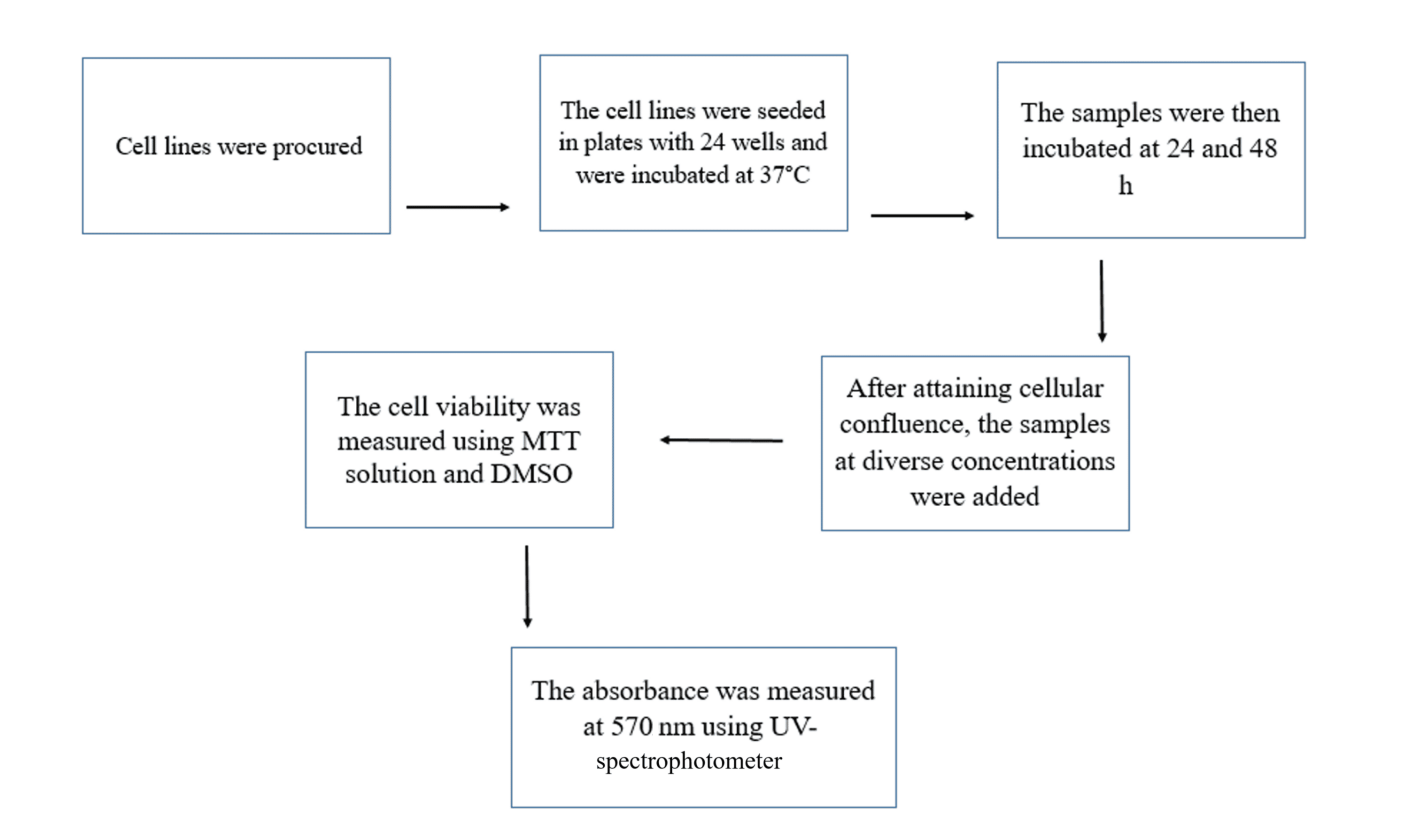
Flowchart representing MTT assay procedure. DMSO: dimethyl sulfoxide; MTT: 3-(4,5-dimethyl-2-yl)-2,5-diphenyltetrazolium bromide

Cells (1 x 10^5^ per well) were seeded in plates with 24 wells and incubated at 37°C with five percent CO_2_. Upon attainment of cellular confluence, the samples at diverse concentrations were added and incubated for 24 h and 48 h. Following the incubation period, the samples were carefully extracted from the wells and rinsed with either phosphate-buffered saline or serum-free DMEM to ensure optimal conditions for the subsequent step. Next, 100 μL/well (5 mg/mL) of a 0.5% solution of 3-(4, 5-dimethylthiazol-2-yl)-2, 5-diphenyltetrazolium bromide, a vital dye, was added and incubated for the duration of 4 h. This tetrazolium-based assay is a powerful tool for evaluating cellular viability and proliferation, as the dye is metabolically reduced by living cells to produce a colored formazan product that can be readily quantified [16]. Subsequent to the incubation period, 1 mL of dimethyl sulfoxide (DMSO) was introduced into each well, effectively dissolving the formazan crystals. This enabled the absorbance to be precisely measured at 570 nm using a UV spectrophotometer, with DMSO serving as the optimal blank reference (Figure 1).

The percentage of cell viability was calculated using the following formula: percentage of cell viability = A570 of treated cells/A570 of control cells × 100, here A is absorbance. Graphical representations display the percentage of cell viability on the Y-axis, with the sample concentration situated on the X-axis. Each assay incorporates both cell control and sample control to enable comprehensive comparisons of cell viability assessments.

## Results

To evaluate the biocompatibility of the herbal consortium on the Vero cell line, an MTT assay was carried out over a 24-48 h period of incubation with seven different concentrations 500 µg, 250 µg, 125 µg, 62.5 µg, 31.2 µg, 15.6 µg, and 7.8 µg. A dose-dependent cell viability was observed. Every experiment was conducted three times.

Assessment of cell viability of herbal consortium on Vero cell line

**Figure 2:**
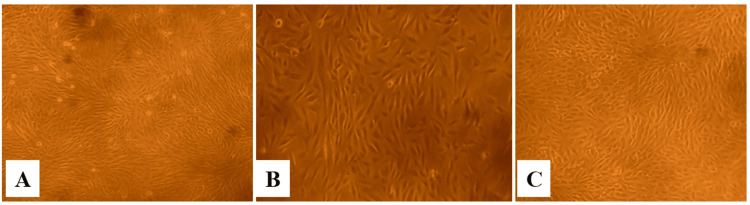
The cytotoxic effect of the herbal consortium at 24 h in the Vero cell line at various concentrations. The images show (A) control, (B) the highest concentration of 1000 µg/mL, and (C) the lowest concentration of 7.8 µg/mL.

At 24 h, the lowest viability was observed at 1000 µg in which 59.38% of the cells were viable, followed by 64.70%, 70.30%, 75.35%, 80.67%, 86.41%, 92.15%, and 97.89% at concentrations 500 µg, 250 µg, 125 µg, 62.5 µg, 31.2 µg, 15.6µg, 7.8µg, and control (untreated cells), respectively (Table 1, Figures 2A-2C).

**Table 1:** Cytotoxicity effect of herbal consortium on Vero cell line (24 h). OD: optical density

S. no.	Concentration (µg/mL)	Absorbance (OD)	Cell viability (%)
1	1000	0.424	59.38
2	500	0.462	64.70
3	250	0.502	70.30
4	125	0.538	75.35
5	62.5	0.576	80.67
6	31.2	0.617	86.41
7	15.6	0.658	92.15
8	7.8	0.699	97.89

**Figure 3:**
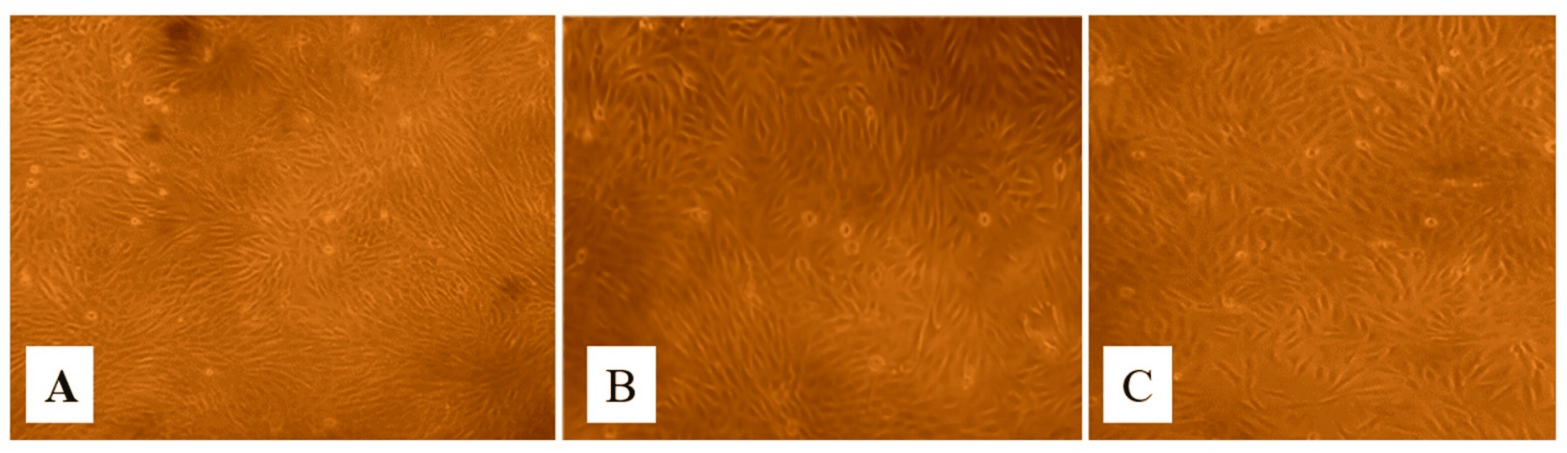
The cytotoxic effects of the herbal consortium at 48 h on the Vero cell line at various concentrations. The images show (A) control, (B) the highest concentration of 1000 µg/mL, and (C) the lowest concentration of 7.8 µg/mL.

At 48 h, lowest viability was observed at 1000 µg at which 68.62% of the cells were viable, followed by 73.08%, 77.55%, 82.01%, 86.47%, 90.81%, 95.28%, and 99.74% at concentrations 73.08 µg, 77.55 µg, 82.01 µg, 86.47 µg, 90.81 µg, 95.28 µg, 99.74 µg, and control (untreated cells), respectively (Table 2, Figures 3A-3C).

**Table 2:** Cytotoxicity effect of herbal consortium on Vero cell line (48 h). OD: optical density

S. no.	Concentration (µg/mL)	Absorbance (OD)	Cell viability (%)
1	1000	0.538	68.62
2	500	0.573	73.08
3	250	0.608	77.55
4	125	0.643	82.01
5	62.5	0.678	86.47
6	31.2	0.712	90.81
7	15.6	0.747	95.28
8	7.8	0.782	99.74

The significant concentration that showed maximum viability was seen at 7.8 µg in both 24 h and 48 h incubation periods which was 97.89% and 99.74%, respectively.

## Discussion

The scientific community recognizes plants as a rich and diverse source of natural compounds with immense therapeutic potential. Plant-derived extracts are attracting increasing attention and interest due to their natural origins, as well as their promising potential for developing effective, safe, and comprehensive healthcare approaches [17]. Plant leaf extracts have emerged as a particularly captivating and promising medical avenue, with substantial potential for managing oral precancerous conditions and promoting accelerated wound healing [18]. The diverse array of plant-derived phytochemicals, including a wide range of antioxidants, anti-inflammatory compounds, and other bioactive molecules have consistently demonstrated extraordinary potential to not only accelerate the wound healing process but also catalyze tissue regeneration and effectively mitigate both inflammation and microbial infections [19,20]. Additionally, plant-derived compounds have been extensively explored for their potent antimicrobial properties, offering a potentially valuable alternative to synthetic antibiotics [21]. However, evaluating the efficacy and safety of traditional herbal remedies can be challenging as they may not easily align with the standard protocols of modern scientific investigation. Additionally, the transfer of a remedy from one country to another may not be straightforward, as factors such as environmental conditions, cultural context, and healthcare systems can all potentially influence the effectiveness of a treatment [22].

One of the key concerns surrounding both conventional and traditional medicine is their potential for cytotoxicity or the ability to cause cell damage or death [23]. Conventional chemotherapeutic agents, for instance, are known to have significant cytotoxic effects, as they often target rapidly dividing cells, including both malignant and healthy cells [24]. This lack of selectivity can lead to severe and unintended side effects, such as loss of appetite, nausea, and damage to vital organs. Similarly, some traditional herbal remedies have also been found to possess potent cytotoxic properties. While certain herbal compounds may be effective in treating specific conditions, their cytotoxic effects can also be harmful, particularly if the patient is unaware of the potential risks or if the remedies are not properly regulated and standardized [25]. Evaluating the cytotoxic effects of herbal extracts is essential to understand their risks and benefits. Assessing the toxicity of these natural compounds is crucial, as it provides insights into their safety and effectiveness for traditional medicine. This analysis is needed to make informed decisions about using herbal remedies.

Ethanolic extraction is a particularly promising method for obtaining these valuable plant-derived compounds, as it is cost-effective, sustainable, and can selectively target and isolate specific bioactive compounds of interest [26,27]. When the diverse array of bioactive compounds from various plant sources are carefully blended and combined, they can interact synergistically to enhance overall therapeutic efficacy and offer a much wider range of medicinal benefits, particularly for complex or multifactorial diseases [28,29]. The Vero cell line is exceptionally well-suited for assessing the biocompatibility and safety of these plant-derived compounds due to its high sensitivity to both microbes and toxins, its well-characterized genome, and its established culturing protocols [30,31]. The use of plant-derived extracts and compounds holds great promise for developing innovative and effective healthcare solutions that leverage the natural therapeutic potential of these diverse plant sources [32].

**Figure 4:**
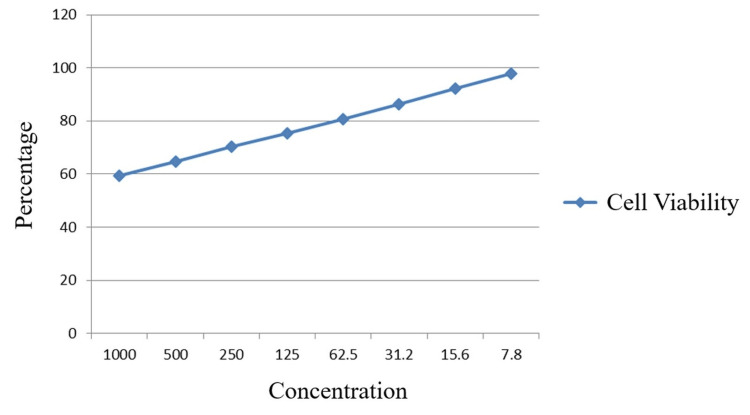
Graph showing decrease in viability of cells with increase in concentration of the herbal consortium (24 h).

**Figure 5:**
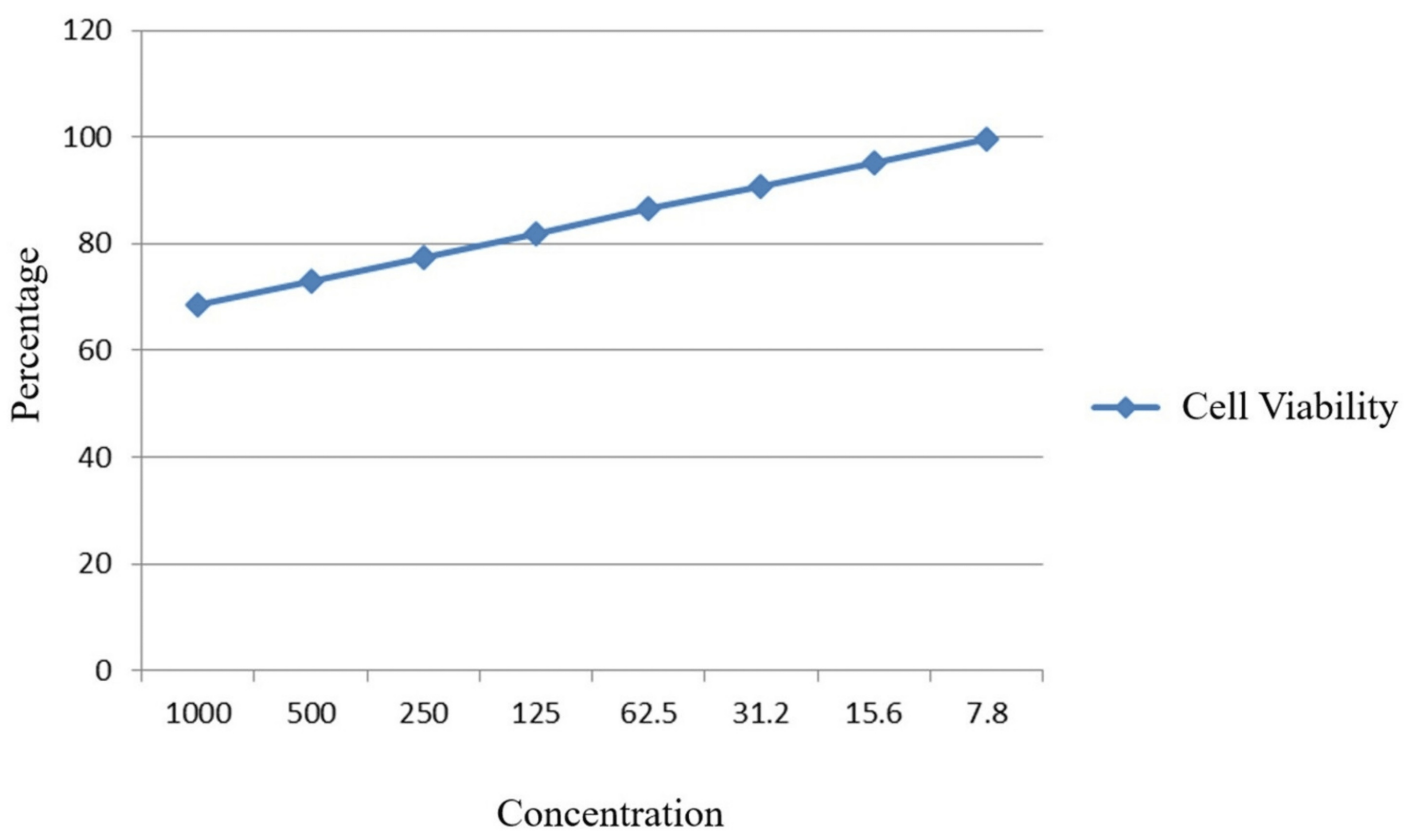
Graph showing decrease in viability of cells with increase in concentration of the herbal consortium (72 h).

Our comprehensive research and expertise have led us to carefully select and blend three esteemed herbal extracts - Vilvam, Nochi, and Adhimadhuram - into a potent herbal elixir. This exceptional botanical formulation has been extensively evaluated for its biocompatibility and therapeutic potential using the Vero cell line, a well-established model system. Remarkably, the herbal elixir exhibited heightened cytotoxicity on the Vero cells, with a corresponding decline in viable cell percentage as the concentration of the formulation was increased (Figures 4, 5).

This profound impact was rigorously examined at both 24 and 48 h time points, wherein the viable cell count was observed to modestly increase over time. The cell viability assay demonstrated a concerning dose-dependent decline in cellular activity, with viability observed to be only 59.38% and 68.62% at the highest tested concentration of 1000 μg/mL after 24 and 48 h, respectively. These findings strongly suggest the remarkable bioactive potential of this botanical formulation, potentially offering a promising natural therapeutic approach for further investigation. The observed cytotoxicity of the herbal elixir on the Vero cell line, a well-characterized non-cancerous cell model, indicates that this formulation may possess potent biological activities that warrant further exploration. Additional studies are needed to fully elucidate the mechanism of action, selectivity, and potential therapeutic applications of this exceptional botanical blend. While comprehensive data on the synergistic effects of this unprecedented herbal elixir are currently unavailable, the promising individual results observed in prior cell-based studies for its component herbs provide a compelling basis for further investigation. Extant research has demonstrated the potent cytotoxic effects of licorice leaf extracts on diverse cancer cell types, implying their promising therapeutic utility in oncology [33]. However, the cytotoxicity of these extracts on normal, non-cancerous cells remains an important consideration that warrants further investigation. These studies have shown that licorice leaf extracts can exert selective cytotoxicity towards cancerous cells while having a relatively lower impact on the viability of normal, healthy cells. This selectivity presents an intriguing opportunity for developing more targeted and effective cancer treatments using plant-derived compounds [34]. Additionally, research has shown that *Vitex negundo* leaves possess potent antibacterial and anticancer effects, with some studies indicating selectivity towards cancer cells while having minimal cytotoxicity on normal cells. This ability to preferentially target and eliminate cancerous cells, while leaving healthy cells relatively unharmed, is a highly desirable trait for potential therapeutic agents [35,36]. Similarly, the *Aegle marmelos *leaf extract has displayed a dose-responsive cytotoxic influence on the Vero cell line, highlighting the potential of this plant-derived compound to exert specific effects on different cell types and potentially offer therapeutic benefits [10]. These findings suggest that the herbal elixir, which combines these potent plant extracts, may hold significant promise as a natural, targeted approach for cancer treatment and management, warranting further comprehensive investigation and evaluation. While the present study's in vitro cell viability assessments offer valuable insights, their limitations underscore the pressing need to harness more advanced molecular techniques, such as polymerase chain reaction (PCR) and enzyme-linked immunosorbent assay (ELISA), to unravel the complexities of these herbal formulations. A comprehensive analysis leveraging a diverse array of sophisticated analytical tools is indispensable to gain a deeper, more nuanced understanding of these therapeutic agents.

## Conclusions

The herbal formulation showcases impressive biocompatibility. Vero cell testing demonstrated the herbal blend's exceptional capacity to support cell viability with negligible toxicity, strongly indicating its suitability for future biomedical applications. However, realizing the full potential of this promising herbal consortium will necessitate extensive further research, including rigorous animal studies and clinical trials to meticulously evaluate its safety and efficacy. A deeper investigation into the underlying biocompatibility mechanisms and therapeutic properties will be pivotal to unlocking the transformative possibilities of this innovative herbal blend.
